# Pancreatic cancer clusters and arsenic-contaminated drinking water wells in Florida

**DOI:** 10.1186/1471-2407-13-111

**Published:** 2013-03-12

**Authors:** Wen Liu-Mares, Jill A MacKinnon, Recinda Sherman, Lora E Fleming, Caio Rocha-Lima, Jennifer J Hu, David J Lee

**Affiliations:** 1 Department of Epidemiology and Public Health, Sylvester Comprehensive Cancer Center, University of Miami Miller School of Medicine, 1120 NW 14th St., CRB 1512, Miami, FL, 33136, USA; 2 Department of Hematology and Oncology, University of Miami Miller School of Medicine, Miami, FL, USA; 3 Florida Center Data System (FCDS), University of Miami Miller School of Medicine, Miami, FL, USA; 4 The European Centre for Environment and Human Health, Peninsula College of Medicine and Dentistry, University of Exeter, Cornwall, UK

**Keywords:** Pancreatic cancer, Screening, Arsenic, Epidemiology

## Abstract

**Background:**

We sought to identify high-risk areas of pancreatic cancer incidence, and determine if clusters of persons diagnosed with pancreatic cancer were more likely to be located near arsenic-contaminated drinking water wells.

**Methods:**

A total of 5,707 arsenic samples were collected from December 2000 to May 2008 by the Florida Department of Health, representing more than 5,000 individual privately owned wells. During that period, 0.010 ppm (10 ppb) or greater arsenic levels in private well water were considered as the threshold based on standard of United States Environmental Protection Agency (EPA). Spatial modeling was applied to pancreatic cancer cases diagnosed between 1998–2002 in Florida (n = 11,405). Multivariable logistic regression was used to determine if sociodemographic indicators, smoking history, and proximity to arsenic-contaminated well sites were associated with residence at the time of pancreatic cancer diagnosis occurring within versus outside a cluster.

**Results:**

Spatial modeling identified 16 clusters in which 22.6% of all pancreatic cancer cases were located. Cases living within 1 mile of known arsenic-contaminated wells were significantly more likely to be diagnosed within a cluster of pancreatic cancers relative to cases living more than 3 miles from known sites (odds ratio = 2.1 [95% CI = 1.9, 2.4]).

**Conclusions:**

Exposure to arsenic-contaminated drinking water wells may be associated with an increased risk of pancreatic cancer. However, case–control studies are needed in order to confirm the findings of this ecological analysis. These cluster areas may be appropriate to evaluate pancreatic cancer risk factors, and to perform targeted screening and prevention studies.

## Background

Pancreatic cancer is one of the most common causes of cancer mortality. The American Cancer Society estimated that 43,140 persons in the US would be diagnosed with pancreatic cancer in 2010, and that 94% of the patients will die from this highly lethal malignancy [1]. Each year 250,000 people worldwide will die of pancreatic cancer [2]. Late diagnosis, lack of therapeutic options, and the aggressive biological nature of pancreatic cancer cells play major roles in the traditionally poor prognosis of pancreatic cancer [3]. Although efforts are being made to understand the initiation and progression of this cancer and to identify the factors that confer its particular aggressiveness, the exact environmental and/or genetic events underlying the development of this malignancy remain undiscovered.

Although the etiology of pancreatic cancer is largely unknown after decades of intensive research, smoking is one of the few factors consistently associated with pancreatic cancer risk. It is estimated that smoking accounts for 20-25% of all pancreatic tumors. People who use smokeless (spit or chew) tobacco are also more likely to develop pancreatic cancer. Previous studies have demonstrated that smokers have a 1.5-3 times increased risk of developing pancreatic cancer [4-15]. In an addition to cigarette smoking, consistent evidence of a positive association has been found between family history and pancreatic cancer. With the exception of tobacco smoking and family history, other risk factors for pancreatic cancer have not been well-established.

Arsenic is linked to bladder, skin, and lung cancer occurrence in populations highly exposed to arsenic occupationally, medicinally, or through exposure to contaminated drinking water [16,17]. Many of the more recent studies linking arsenic exposure to these cancer outcomes were conducted in countries outside of the US, such as Scandinavian countries [18,19], Taiwan [20-23], Argentina [24] and Chile [25]. In this latter study, odds of lung cancer increased in a dose–response fashion with increasing exposure to arsenic-contaminated drinking water. Relative to those with low exposure (mean urinary arsenic level < 9 ug/l), the odds of lung cancer in the highest exposure category (mean urinary concentration = 825 ug/l) was 7.1 (3.4-14.8) [25]. Significant elevated risk was observed at mean urinary concentrations as low as 126.1 ug/l (OR = 3.4; 95% CI = 1.8-6.5). In the study conducted in Argentina, ingested arsenic was associated with a significant increased risk of bladder cancer in smokers but not among nonsmokers (2.17; 1.02-4.63) [24].

More recent US-based studies have examined associations between arsenic exposure and bladder, skin, and lung cancers [26-28]. In a study of arsenic-contaminated drinking water wells in New Hampshire, there was an elevated but non-significant odds ratio for bladder cancer for the uppermost category of arsenic exposure as determined by toenail analysis among ever smokers (2.17; 0.92-5.11); there was no evidence of an increased cancer risk in never smokers irrespective of arsenic exposure levels [26]. In another analysis conducted in this state, those in the most extreme exposure category (>97th percentile) had an age and gender adjusted odds ratio for squamous cell carcinoma of 2.07 (0.92- 4.66) [27]. Finally, a case–control study drawn from residents of New Hampshire and Vermont found that arsenic exposure was associated with risk of small-cell and squamous-cell carcinoma of the lung (2.75; 1.0- 7.57) among those with toenail arsenic concentration > 0.114 ug/g versus < 0.05 ug/g [28]. In Florida, clusters of bladder cancer were found among those who live in close proximity to known arsenic-contaminated drinking water wells [29].

In contrast to the research on bladder, skin, and lung cancers, there appears to be no consistent association between arsenic exposure and pancreatic cancer [30] and virtually no recent research on this topic in the US. However, as indicated above arsenic may have a role as a co-carcinogen when paired with other carcinogens such as smoking [24,26]. Over the past five years, pancreatic cancer has been one of the few invasive malignancies that have been rising in Florida 2002–2006, and the mortality rate of this fatal cancer has not changed (http://www.cdc.gov/cancer/npcr/). In this study, we sought to ascertain if there were any pancreatic cancer clusters in Florida, and to identify socio-demographic and behavioral correlates associated with these clusters. Controlling for these factors, we also explored if pancreatic cancer cluster membership was associated with proximity to identified arsenic-contaminated drinking water wells.

## Methods

### Overview

Florida residents diagnosed with pancreatic cancer between 1998 and 2002 were identified by the State of Florida incidence cancer registry, the Florida Cancer Data System (FCDS). The International Classification of Diseases (ICD) – Oncology, 3rd edition was used to code primary site and morphology (site code C25.0 through C25.9 and all morphologies). Residence at the time of diagnosis was recorded and geocoded for spatial analysis at the census block-group level. The block group was chosen because it is the smallest geographic unit for which US census data are available. It allows more precise socioeconomic status assignment than the census tract or zip code. This study was approved by the University of Miami and the Florida Department of Health Institutional Review Boards.

#### Cancer registry data

The Florida Cancer Data System (http://fcds.med.miami.edu/) (FCDS) has collected incident cancer data since 1981. FCDS is part of the National Program of Cancer Registries (NPCR), which is administered by the Centers for Disease Control (CDC). Cancer incidence data are submitted to the FCDS from all hospitals, laboratories, ambulatory surgical centers, and radiation therapy centers in Florida. There are approximately 115,000 newly diagnosed cancer cases per year among Florida’s 17.5 million residents in 67 counties. At present, the FCDS database contains more than 2.7 million cancer incidence records.

#### Health data

Aggregated patient data were the numerator data; the aggregated 2000 census population (multiplied by five to reflect the number of incident years and also stratified by gender and age group) was the denominator data for the spatial analyses. We utilized spatial analysis to identify the clusters of block groups with higher than expected pancreatic cancer incidence, and logistic regression analysis to model the probability of pancreatic cancer cases falling within and outside of these geographic clusters at the time of diagnosis as a function of socioeconomic status, reported tobacco use, and proximity to known arsenic-contaminated wells.

The dependent variable was the block group assignment of having an excess incidence of pancreatic cancer versus an expected or lower pancreatic cancer incidence using cluster detection software (as described below). The independent variables were patient (i.e. FCDS derived data) and area-based (census derived) measures, as well as the distance between the residence at time of diagnosis and arsenic-contaminated wells as documented by the Florida Department of Health; race/ethnic categories; census-derived poverty status at the block-group level; and census-derived county-level urban/rural residence. Smoking status provided by patient self-report was obtained from the medical record at the time of pancreatic cancer diagnosis in the FCDS record.

#### Arsenic contamination data

The detailed procedure of collecting arsenic contamination data has been described previously [29]. Arsenic data were provided by the Florida Department of Health Drinking Water Toxics Program, a non-regulatory program responsible for coordinating groundwater sampling for chemical contamination of private drinking water supplies throughout Florida. A total of 5,707 arsenic samples were collected from December 2000 to May 2008 by the Florida Department of Health, representing more than 5,000 individual privately owned wells that were tested for arsenic. During that period, 551 samples were detected with 0.010 ppm (10 ppb) or greater arsenic levels in private well water. For study purposes, we considered 10 ppb as the threshold since during this period the United States Environmental Protection Agency (EPA) changed the standard for arsenic from 50 to 10 ppb in public water systems. Although private wells are considered non-regulatory, the Florida Department of Health recognizes the EPA standard as the maximum containment level in drinking water.

The distance between patient residences and contaminated drinking water wells was calculated in miles using the ArcGIS™, version 9.0 buffering feature. For all block groups in the respective buffers, the patient record was annotated with the number of miles. Results were categorized into greater than three miles, between greater than one and three miles or less, and less than or equal to 1 mile from a known arsenic-contaminated drinking water well.

#### Spatial and statistical analysis

ArcGIS, version 9.0 was the geographic information system used for this analysis to view, analyze, calculate distance and relate data from a spatial (geographic) perspective. SaTScan™, version 5.0, was used to identify block groups in Florida with excess pancreatic cancer [31,32]. FCDS pancreatic cancer data were aggregated at the block group level by gender and age group, and served as SaTScan numerators. Using Monte Carlo techniques, SaTScan assigned relative risk probabilities to defined block groups to detect both the location of clusters and evaluate their statistical significance. Race and the standard age (18 years) groups were used as covariates in this analysis.

Under the null hypothesis, the incidence of pancreatic cancer follows a Poisson distribution, and the probability of a case being diagnosed in a particular location is proportional to the covariate-adjusted population in the location. For hypothesis testing, the SaTScan program generated 999 random replications of the data set under the null hypothesis. The test statistic was calculated for each random replication as well as for the real data set. When the latter was among the 5% highest, the test was significant at the 0.05 level [32]. Multiple testing of cluster locations and sizes was adjusted in analyses of the spatial scan statistic [33,34].

**Figure 1:**
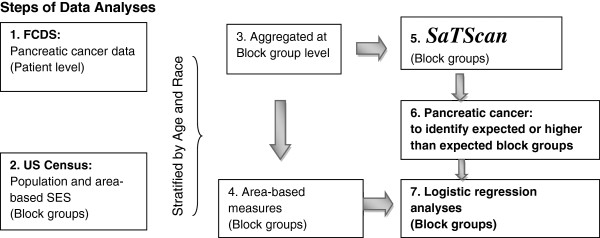
Steps of Data Analyses.

Multivariable logistic regression with SPSS®, version 11.0.1, was performed to assess potential predictor variables across groups. In these models, the dependent variable was a patient with pancreatic cancer living in a neighborhood (“block groups”) with a higher than expected pancreatic cancer incidence (a “cluster”) versus not being diagnosed in a cluster. Gender and age were not part of the multivariate logistic regression models because they were already incorporated as covariates in the SaTScan analysis to identify areas of higher than expected incidence. Reported odds ratios (OR) and 95% confidence intervals (CI) are adjusted for all model covariates (e.g. distance to a contaminated well, race/ethnicity, urban/rural location, and tobacco use). Figure 1 shows the steps of these analyses.

## Results

From 1998 through 2002 in Florida, 11,405 patients 13 to 104 years old (median age 73.0) were diagnosed with pancreatic cancer (Table 1). The majority of cases were white, non-Hispanic (81.3%). Most of the diagnosed cases lived more than three miles from known arsenic-contaminated wells (90.6%), although nearly 3% of cases were living less than one mile from known arsenic-contaminated wells at the time of diagnosis.

**Table 1:** Characteristics of Florida pancreatic cancer cases, 1998-2002

	**Number of pancreatic cases (%)**
**Total number of cases**	**11,405**
**Proximity to arsenic- contaminated wells**	
**>3 miles**	10330 (90.6%)
**1-3 miles**	746 (6.5%)
**<= 1 mile**	329 (2.9%)
**Poverty status**	
**Non Poverty**	8,572 (75.2%)
**Poverty**	1,951 (17.1%)
**Unspecified**	882 (7.7%)
**Race/ethnicity**	
**Non-Hispanic white**	9271 (81.3%)
**African-American**	956 (8.4%)
**Hispanic**	1043 (9.1%)
**Unspecified**	135 (1.2%)
**Gender**	
**Male**	5718 (50.2%)
**Female**	5681 (49.8%)
**Unspecified**	6 (0.0%)
**Tobacco use**	
**Never**	3880 (34.0%)
**Current smoker**	2671 (23.4%)
**Former smoker**	1791 (15.7%)
**Other/Unknown**	3063 (26.9%)
**Pancreatic cancer cases**	
Clustered*	2,581 (22.6%)
**Non-Clustered**	8,824 (77.4%)

*Based on 16 identified clusters.

### Spatial analysis results

**Figure 2:**
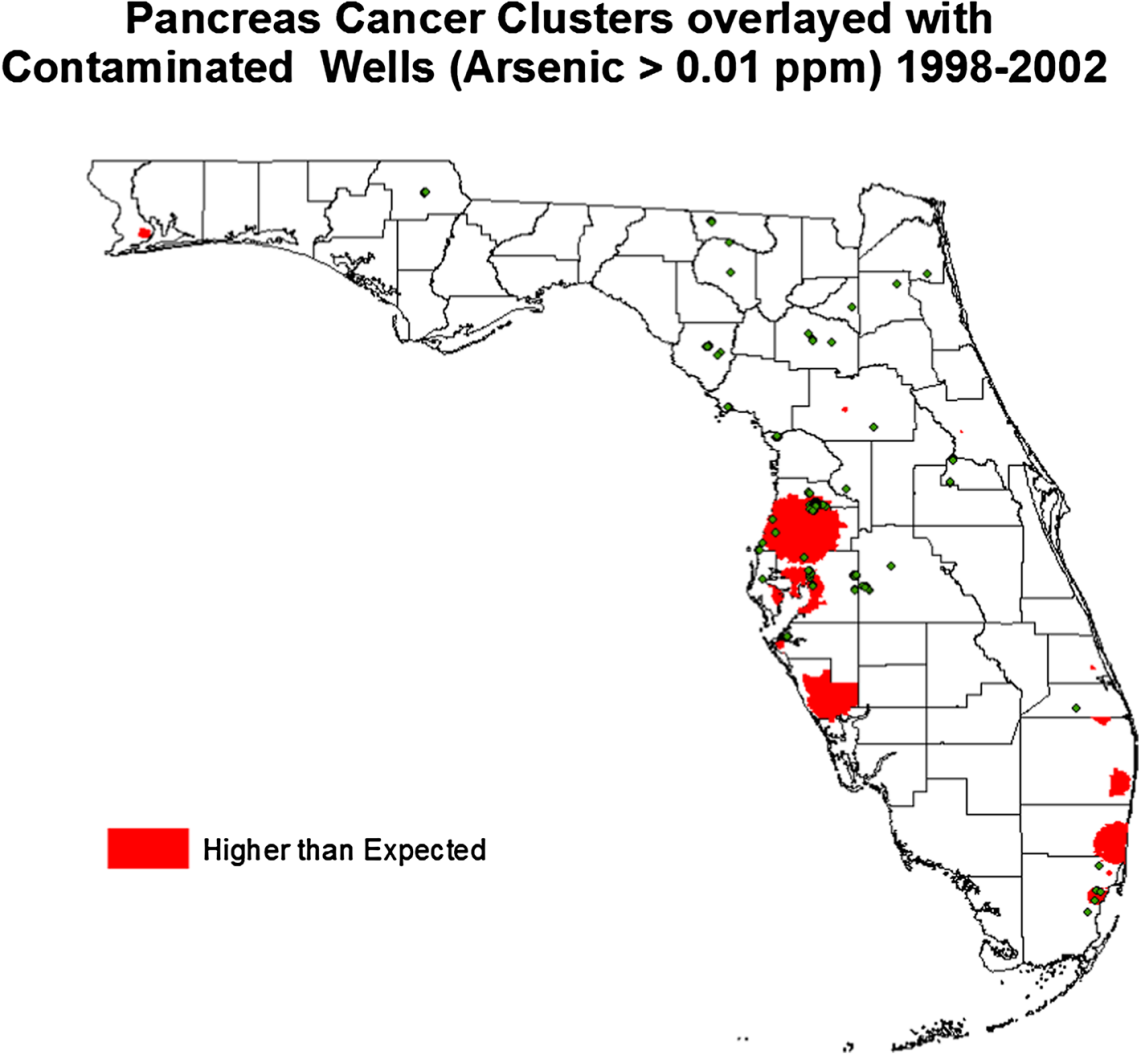
Pancreas Cancer Clusters overlaid with Arsenic Contaminated Wells in Florida 1998–2002: green circles represent arsenic contaminated wells and red areas represent higher than expected pancreatic cancer clusters.

During the study period, patients with pancreatic cancer lived in 1753 of the 9112 block groups in Florida. There were 16 clusters identified with a higher than expected pancreatic cancer rate. This represented 2619 patients or 23.0% of all the pancreatic cancer cases (Table 1). Figure 2 depicts these clusters and the locations of arsenic-contaminated wells in Florida. Clusters tended to be located on the Eastern and Western coasts below the “Panhandle” region of the state.

### Multivariable logistic regression analyses

After adjustment for sociodemographic and smoking status, pancreatic cancer cases were more likely to be in areas of a higher than expected incidence when living near a drinking water well known to contain arsenic (Table 2). Cases living within 1 mile of known arsenic-contaminated wells were significantly more likely to be diagnosed within a cluster of pancreatic cancers relative to cases living more than 3 miles from known sites (OR = 2.1; 95% CI = 1.9- 2.4); on the other hand, compared to cases living more than 3 miles from known sites, there was no increased risk for those living within 1–3 miles of a known site (1.0; 0.8- 1.2).

**Table 2:** Multivariable-adjusted correlates of living within versus outside of one of 16 pancreatic cancer clusters at the time of diagnosis, Florida 1998–2002 (n = 11,405)

	**Odds ratios (95% CI)**
**Proximity to arsenic contaminated well water**	
>3 miles	1.0
1-3 miles	1.0 (0.8, 1.2)
<= 1 mile	**2.1 (1.9, 2.4)**
**Social economic status**	
Non poverty	1.0
Poverty	1.1 (0.9, 1.2)
**Tobacco use**	
Never	1.0
Current smoker	**1.1 (1.0, 1.2)**
Former smoker	**0.9 (0.8, 1.0)**
Other/unknown	**1.1 (1.0, 1.3)**
**Race/ethnicity**	
Non-Hispanic white	1.0
African-American	**1.6 (1.3, 1.8)**
Hispanic	**0.7 (0.6, 0.8)**

Poverty status was not associated with being diagnosed outside versus inside a pancreatic cancer cluster. Self-reported tobacco use at diagnosis was slightly, but significantly associated with being diagnosed within versus outside a pancreatic cancer cluster compared with non-smokers (1.1; 1.0-1.2). There was also a slightly reduced risk of diagnosis within a pancreatic cancer cluster among former smokers relative to non-smokers (0.9; 0.8-1.0). Compared to Caucasians, there was a significantly increased risk of diagnosis within versus outside of a pancreatic cancer cluster among African- Americans (1.6; 1.3-1.8), with an opposite pattern noted for Hispanics relative to Caucasians (0.7; 0.6- 0.8).

## Discussion

In our study, we identified 16 specific areas in Florida where pancreatic cancer cases were significantly higher than expected. These areas tended to be in urban as opposed to rural communities. Furthermore, we found an association between relatively close proximity to arsenic-contaminated drinking water wells and clusters of pancreatic cancer. Specifically, we found that living within 1 mile of the known arsenic-contaminated drinking water well might be a threshold distance for an increased risk of being diagnosed within a cluster. Community poverty status was unrelated to cancer cluster membership, but we did find that African-American Floridians diagnosed with pancreatic cancer were more likely to be diagnosed within versus outside of these 16 cancer clusters.

As discussed above, the etiology of pancreatic cancer is largely unknown after decades of studying this highly fatal disease. Smoking is one of the few factors found consistently associated with pancreatic cancer. Differences in exposure to tobacco smoke across communities in Florida could partially explain the presence of pancreatic cancer clusters, although we were unable to fully test this hypothesis given that we were limited to data on smoking status collected at the time of cancer diagnosis.

In animal models, low concentrations of arsenic exposures alone do not cause cancers. However, the synergistic effects of arsenic and other carcinogens (such as smoking and ultraviolet irradiation) are suggested to enhance the tumorigenicity [35]. Drinking water arsenic exposure has been associated with increased bladder cancer susceptibility. The findings of an increased bladder cancer risk among smokers but not among nonsmokers by Steinmaus et al. [24], and Karagas et al. [26], suggest that the ingestion of low to moderate arsenic levels may affect bladder cancer incidence, and that cigarette smoking may act as a co-carcinogen as a DNA damaging agent. These findings of synergistic effects of arsenic and other carcinogens (such as smoking) in bladder cancer risk may indicate a similar mechanism for the development of pancreatic cancer.

The biologic effects of arsenic exposure on cancer risk remains largely unknown. The carcinogenic mechanisms of arsenic-induced cancers might be through blocking DNA repair, stimulating angiogenesis, altering DNA methylation patterns, dysregulating cell cycle control, induction of aneuploidy, and/or blocking apoptosis [35,36]. Additional pathways suggested include: endocrine disruption, suppression of hormone regulation and hormone mediated gene transcription, alteration of cell cycle kinetics, and alterations in cellular proliferative response that might play roles in association with carcinogenesis [37]. Recent evidence further supports the hypothesis that sources of DNA damage may interact with arsenic to induce tumorigenesis. For example, arsenic gene-environment interactions have been identified in several studies [38-40].

While most of these studies have studied other cancers instead of pancreatic cancer, and have identified important adverse health effects associated with relatively high-dose arsenic exposure, results cannot be extrapolated to US populations which are typically exposed to lower levels of arsenic exposure. Additionally, precise measurement of exposure is critical to assessing risk in populations consuming relatively trace amounts of arsenic. Other factors may also add uncertainties to the results. For example, not all arsenic exposure studies take exposure to other carcinogens into account (e.g., cigarette smoking). Another possible limitation is the utilization of US Environmental Protection Agency (EPA)’s models to evaluate pancreatic cancer risk, which may underestimate personal cancer risks for arsenic [41].

### Study limitations

The population data were extrapolated for the 5-year study period by multiplying the single 2000 census year population by 5 to estimate the denominator data for spatial analysis which could magnify any inaccuracies in the data. Smoking status was abstracted from the medical record at the time of cancer diagnosis, which may be inaccurate. Furthermore, over one fourth of patients (26.9%) did not report their smoking status. Therefore, misclassification of this important risk factor is likely present.

The results of the study may also be influenced by the completeness and quality of the geocoded data. For this study, approximately 5% of the pancreatic cases were excluded from the analysis because their reported residential address at diagnosis could not be geocoded. Two percent of the cases were included in the analysis, but were geocoded to the centroid of the zip code based on a PO Box address. These cases may not have lived in the same zip code where their PO Box is located at time of diagnosis. The ungeocodeable cases were slightly older (mean age of 72 versus 71) and had a greater percentage of cases reported at time of death instead of at diagnosis (13% versus 10%). Conversely, the cases geocoded based on PO Box were younger (mean age of 68 versus 71) and less likely to be reported to the cancer registry at time of death (1% versus 10%).

Private non-regulatory drinking water well testing for arsenic and other chemicals in Florida is not universal. Samples are usually collected by county health departments in response to citizen complaints or suspicion of potential contamination by an anthropogenic source (e.g., previous agricultural land use). This nonsystematic ad hoc sampling approach may not accurately identify or capture where actual concentrations of arsenic may be in the soil and water. Furthermore, we only had access to the patient residence at diagnosis and had no information on prior residence history in patients with pancreatic cancer, which is important, given the 15 to 30-year pancreatic cancer latency. The other limitation of the study pertains to the accuracy of exposure assessment since arsenic levels in the well water may not reflect individual exposure. While we cannot accurately ascertain individual well water exposure, in Florida there are more than 1.5 million private wells from which approximately 20% of the population receives water (http://www.doh.state.fl.us/environment/water/privatewells.html). If arsenic is in the soil and air, then arsenic could be dissolved from the arsenic-bearing soil and air into the ground water (particularly given the heavy rainfall experienced in Florida). Therefore, well water may serve as a proxy for environmental arsenic contamination in the soil and air.

Despite these limitations, we believe that our study represents the first step in identifying communities that may benefit from further investigation. Most of previous studies in literature were done in other countries, with much higher exposure sources. Our criteria for exposure was likely much lower in our study. Our study suggests that these ‘lower’ exposure levels may be important. The combined and possibly synergistic effects of arsenics and other carcinogens such as smoking and ultraviolet irradiation may enhance the tumorigenicity [35]. Factors which could account for our ability to detect these low-level exposures could be the completeness of cancer case registry in Florida, the accuracy of the census-derived denominator data, the geocoded cases, and the sensitivity of the SatScan program.

## Conclusions

Arsenic exposure in drinking water has been associated with increased bladder and lung cancer susceptibility. Epidemiologic and experimental data suggest a co-carcinogenic effect of arsenic with exposure to DNA damaging agents, such as cigarette smoke. The findings of an increased bladder cancer risk among smokers, but not among nonsmokers by Steinmaus et al. [24] and Karagas et al. [26] suggest further investigations of arsenic, and its interaction with smoking in the development of cancers. However, as the current report is an ecological analysis subject to numerous potential biases it is important to note that future case–control studies are required to investigate the effect of arsenic exposure on pancreatic cancer risk, and its interactions with other co-carcinogens such as smoking in the development of pancreatic cancer.

Future studies to extrapolate cancer risks incorporating the genetic, environmental factors, and their interactive relationship to understand the etiology of pancreatic cancer, and to identify high-risk populations for primary prevention of this deadly disease will be an important endeavor to contribute to reduce the burden of the disease and to promote public health.

There is a public health and clinical imperative to identify the etiology of pancreatic cancer including environmental factors, and to establish foundations for further developing effective and targeted pancreatic cancer prevention programs. The present analysis identified multiple areas of pancreatic cancer clustering within Florida. Furthermore, we identified pancreatic cancer clusters that had an increased likelihood of being located near known arsenic-contaminated wells. If these results are confirmed using more robust study designs, the targeted public awareness campaigns are needed in these high risk communities in order to educate residents on ways to reduce environmental and non-environmental exposures to cancer causing agents, especially the need to reduce community smoking rates.

## Consent

Written informed consent was obtained from the patient for publication of this report and any accompanying images.

## Competing interests

The authors declare that they have no competing interests.

## Authors’ contributions

DJL, LEF, JAM and WLM have been involved in study design and interpretation of the data. DJL, JJH and CRL contributed to the conception of the project. JAM and RS participated in acquisition of the data and carried out the statistical analyses, WLM drafted the manuscript. All authors critically reviewed and approved the final manuscript.

## Pre-publication history

The pre-publication history for this paper can be accessed here:

http://www.biomedcentral.com/1471-2407/13/111/prepub
